# Anatomical Variations of the Pectoralis Major Muscle: Notes on Their Impact on Pectoral Nerve Innervation Patterns and Discussion on Their Clinical Relevance

**DOI:** 10.1155/2019/6212039

**Published:** 2019-04-02

**Authors:** Robert Haładaj, Grzegorz Wysiadecki, Edward Clarke, Michał Polguj, Mirosław Topol

**Affiliations:** ^1^Department of Normal and Clinical Anatomy, Interfaculty Chair of Anatomy and Histology, Medical University of Lodz, ul. Żeligowskiego 7/9, 90-752 Łódź, Poland; ^2^Department of Angiology, Interfaculty Chair of Anatomy and Histology, Medical University of Lodz, ul. Żeligowskiego 7/9, 90-752 Łódź, Poland

## Abstract

**Background:**

The presented study attempts to classify individual anatomical variants of the pectoralis major muscle (PM), including rare and unusual findings. Rare cases of muscular anomalies involving the PM or its tendon have been presented. An attempt has also been made to determine whether anatomical variations of the PM may affect the innervation pattern of the lateral and medial pectoral nerves.

**Material and Methods:**

The research was carried out on 40 cadavers of both sexes (22 males, 18 females), owing to which 80 PM specimens were examined.

**Results:**

Typical PM structure was observed in 63.75% of specimens. The most frequently observed variation was a separate clavicular portion of the PM. In one female cadaver (2.5% of specimens) the hypotrophy of the clavicular portion of the PM was noticed. In two male cadavers (5% of specimens) the fusion between the clavicular portion of the PM and the deltoid muscle was observed. In one of those cadavers, small sub-branches of the lateral pectoral nerve bilaterally joined the clavicular portion of the deltoid muscle. The detailed intramuscular distribution of certain nerve sub-branches was visualized by Sihler's stain. PM is mainly innervated by the lateral pectoral nerve. In all specimens stained by Sihler's technique, the contribution of the intercostal nerves in PM innervation was confirmed.

**Conclusions:**

Surgeons should be aware of anatomic variations of the PM both in planning and in conducting surgeries of the pectoral region.

## 1. Introduction

The pectoralis major (PM) is a large, fan-shaped muscle, typically composed of a clavicular, sternocostal, and abdominal part. The three parts of the PM are attached to the anterior aspect of the medial half of the clavicle, the anterior part of the sternum, and the cartilages of all the true ribs (attachment to the first and/or seventh costal cartilage is often omitted) and to the aponeurosis of the external oblique, respectively [1]. The clavicular portion of the PM is usually separated from the sternocostal portion of the PM by a slight cleft. The insertion of the muscle is located on the lateral lip of the intertubercular sulcus of the humerus [1, 2].

The PM plays an important role in the upper limb movements, especially during adduction and the medial rotation of the arm [3–5]. Due to its relationship to the chest wall and breast, the PM can be considered as one of the key anatomical structures in plastic and reconstructive surgery [6–8]. The importance of PM in orthopedic surgery refers, among others, to the deltopectoral approach [9–12] or to the repair of PM injuries [6, 13–15]. At the same time, the PM belongs to muscles demonstrating high anatomical variability, which may affect performing imaging-based evaluation and understanding the injury findings [4, 16]. Moreover, anatomical variations (especially those related to attachments or unusual muscle morphology) may affect significantly the course of surgical procedures [13, 17].

Anatomical variations of the pectoralis major are often. According to Bergmann et al. [2], all parts of the PM may be more or less separable. The clavicular head of the PM may extend laterally on the clavicle as far as the deltoid muscle and may be fused with it. The sternal and costal heads may be absent or the whole muscle may be absent in rare cases. However, in the medical literature anatomical variations of the PM are found mainly in the form of scattered descriptions of specific anatomical variations, i.e., case reports or case-series reports [18–31]. The only source in which detailed classification of muscles variations was proposed is the text of Perrin [32] from 1871. In the present study, the classification of Perrin [32] was supplemented with information on the percentage of each type of anatomical variation of the pectoralis major muscle, which fills the “gap” in the literature.

The presented study attempts to classify individual anatomical variants of PM, including rare and unusual findings. Particular attention has been paid to the variability of attachments and the variability in the shape of the clavicular portion of this muscle. Furthermore, rare cases of muscular anomalies involving the PM or its tendon have been presented. An attempt has also been made to determine whether anatomical variations of the PM may affect the innervation pattern of the lateral and medial pectoral nerves. The detailed intramuscular innervation pattern of PM was also examined in this study using Sihler's staining technique.

## 2. Material and Methods

The research was carried out on 40 cadavers of both sexes (22 males, 18 females), owing to which 80 PM specimens were examined. The mean age of the cadavers was 69.3 ± 11.8 years (range: 48-90 years), 69.6 ± 13.8 years (range: 48-85 years) of male and 69.1 ± 10.9 years (range: 53-90 years) of female cadavers. The study was approved by the local Bioethics Committee (No: RNN/231/15/KE).

Prior to the qualification of the cadavers for the research, the specimens with scars, traces of trauma, or deformations within pectoral, shoulder, and brachial regions were excluded. The procedure involved exposure of the PM to visualize its morphology. At this stage, the observed anatomical variations of the PM were evaluated. Morphometric measurements were also made using electronic caliper (Mitutoyo, Kanagawa, Japan). The width of the origins of clavicular and sternocostal portions, the width of the PM insertion, and the width of the PM in the midclavicular line were measured. The distance was also measured between the top of greater tubercle of the humerus and the upper border of PM tendon. The degree of asymmetry between the right and left side was assessed for the selected indices (i.e., percentage of total length of the clavicle covered by the origin of the clavicular part of the PM and width of the PM in the midclavicular line). The assumption was made that the degree of asymmetry represents the percentage difference of the value of a given measurement or index between both sides; it shows the percentage difference between the measurement with a larger value and measurement with a smaller value (the degree of asymmetry = measurement with a larger value ÷ measurement with a smaller value × 100%). Standard descriptive statistics were used to summarize the collected data. The Chi-square test was applied to assess differences in the prevalence of anatomical variations between the sexes.

During the further stage of the dissection, the insertion of the PM was cut along the lateral lip of the intertubercular sulcus and reflected to expose the neurovascular bundles and attachment to the costal cartilages. Careful dissection of the neurovascular bundles was performed in accordance with previously described anatomic dissection techniques [33–35]. This stage allowed observing possible differences in PM innervation depending on its anatomical variations. Distances from both the margin of the sternum (parasternal line) and inferior border of the clavicle to the entry points of the neurovascular pedicles within the pectoralis major muscle were also measured at this stage of the procedure. Furthermore, five randomly selected muscles were examined using Sihler's whole mount nerve staining technique [36]. This allowed for the evaluation of a detailed PM intramuscular innervation pattern. The procedure was modified, based on our earlier experience, for a large muscle mass [37]. The initial phase of Sihler's Stain (i.e., maceration and depigmentation) was extended to over 5 weeks due to the large mass of PM. During the last stage (i.e., destaining), a lower concentration of acetic acid in Sihler's solution I was used to better control the destaining process (glacial acetic acid : glycerin : 1% aqueous chloral hydrate = 0.5 : 1 : 6).

## 3. Results

### 3.1. Major Anatomical Variations of the Pectoralis Major Muscle

**Figure 1:**
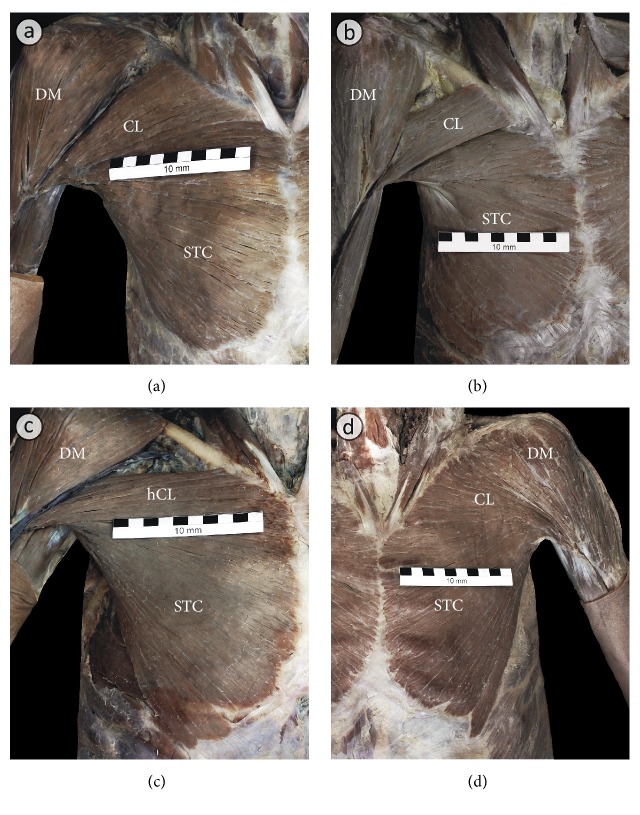
The main anatomical variations related to morphology of the pectoralis major muscle. (a) Typical morphology of the pectoralis major muscle. (b) A separate clavicular part (CL) of the pectoralis major muscle. In these cases, a distinct cleft is visible between the clavicular (CL) and sternocostal portion (STC) of the pectoralis major muscle. (c) The hypotrophy of the clavicular part (hCL) of the pectoralis major muscle. (d) Fusion between the clavicular part (CL) of the pectoralis major muscle and the deltoid muscle (DM). The deltopectoral groove is absent and there is no visible borderline between clavicular portion (CL) of the pectoralis major muscle and the deltoid muscle (DM). The brachial segment of the cephalic vein is absent.

Several anatomical variations related to PM morphology were observed in the examined specimens (Figure 1). The incidence of different types of anatomical variations of the pectoralis major muscle is presented in Table 1. Typical PM structure (Figure 1(a)) was observed in 51 specimens (63.75%), bilaterally in 13 male and 12 female cadavers, and unilaterally in one male cadaver. The most frequent variation was a separate clavicular portion of PM. In these cases, a distinct cleft occurred between the clavicular and sternocostal portion of PM. The degree of separation of these two portions varied in different specimens from partial to almost total (Figure 1(b)). This variation was seen bilaterally in 22 specimens (27.5%) and included six male and five female cadavers.

**Figure 2:**
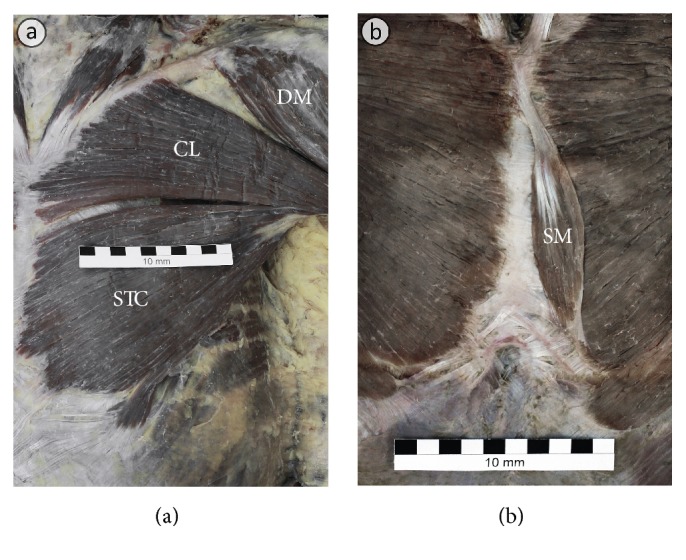
Anatomical variations related to the pectoralis major muscle. (a) Atypical division of PM into two almost completely separate portions is visible at the level of the sternal angle. (b) Presence of the sternalis muscle (SM). CL: clavicular part of the pectoralis major muscle; DM: deltoid muscle; STC: sternocostal part of the pectoralis major muscle.

In one male cadaver, the atypical division of the PM into two almost completely separate portions was present on the left side at the level of the sternal angle (1 out of 80 specimens = 1.25%; Figure 2(a)). In this case the clavicular portion of PM was fused with upper fibers (attached to the manubrium of the sternum) of the sternocostal portion of the PM, forming the upper head of the muscle. The fibers of the sternocostal portion of the PM attached to the body of the sternum formed the lower head of the muscle. A deep cleft was observed between the two heads (the branches of the lateral and medial pectoral nerves were present in the floor of the cleft). The abdominal part of PM was well developed in this case. The morphology of PM on the contralateral side of this male cadaver was typical.

In one female cadaver (2 out of 80 = 2.5% of specimens) the hypotrophy of the clavicular portion of PM was observed (Figure 1(c)). On the left side of the same cadaver the unilateral presence of the sternalis muscle was noted (Figure 2(b)). In two male cadavers (4 out of 80 = 5% of specimens) fusion between the clavicular portion of PM and the deltoid muscle was observed (Figure 1(d)). In the first cadaver, the deltopectoral groove was absent and there was no visible borderline between clavicular portions of the pectoralis major and the deltoid muscles (Figure 1(d)). In this case, the brachial segment of the cephalic vein was absent bilaterally. In the second cadaver, the fusion between the clavicular portion of PM and the deltoid muscle was bilaterally partial with the deltopectoral groove slightly marked. However, the deep fibers of the clavicular portions of the pectoralis major and the deltoid muscles were fused along the whole length. On both sides of the described body the cephalic vein had a typical course; however its terminal segment pierced the muscle fibers to empty into the axillary vein. Thus, the fusion between PM and the deltoid muscle was complete in two specimens of PM (2.5%) and partial also in two specimens of PM (2.5%).

### 3.2. Variations of the Attachments of the PM

Anatomical variations of the origin of PM concerned three main aspects: width of the clavicular part, width of the sternal insertion, and differences in number of costal cartilages involved in PM attachment. Morphometric characteristics of the anatomical variations of the clavicular part of PM are presented in Table 2. The width of the origin of the clavicular portion of PM ranged from 42.5% to 79.2% of total length of the clavicle (mean = 57% ± 11%). On the other hand, in specimens with a separate clavicular portion of the PM, the width of the origin of the clavicular portion of the muscle ranged from 31.9% to 56.4% of the total length of the clavicle (mean= 43.5 ± 7.6%). In contrast, in a cadaver with a poorly developed, hypotrophic part of the clavicular portion of PM, it occupied 26% of the total length of the clavicle on the right side and 22.5% of the total length of the clavicle on the left side. In the case of PM fusion with the deltoid muscle, the width of the clavicular portion of PM ranged from 60.1% to 83.3% of the overall length of the clavicle (mean = 71.5% ± 12.6%). Asymmetry in percentage of the total length of the clavicle covered by the origin of the clavicular part of PM, observed between the right and left side, ranged for all variations from 0.9% to 69.9% (Table 2). The width of the base of the deltopectoral triangle (i.e., the distance between origins of the clavicular portions of PM and the deltoid muscle) varied from 10.3 to 52.9 mm (Table 2). The width of the attachment of the sternocostal portion of PM ranged from 133 mm to 215 mm (mean = 172 mm ± 22 mm; Table 3). Asymmetry in the width of the PM in the midclavicular line assessed for all variations ranged from 1.4% to 17.9% (mean = 6.9% ± 5.2%; Table 3).

The attachments to the costal cartilages were also highly variable. Most frequently, the PM originated from the 2nd to 6th costal cartilages. This type of origin was observed in 58.75% of specimens (47 of 80 specimens; bilaterally in 11 male cadavers, unilaterally in one male cadaver, and bilaterally in 11 female cadavers). In 21.25% of specimens, the PM originated from the 1st to 6th costal cartilages (17 of 80 specimens; in 4 male and 3 female cadavers bilaterally, in 2 male cadavers and 1 female cadaver unilaterally). In 7.5% of specimens, the PM originated from the 2nd to 7th (6 of 80 specimens; in 2 male cadavers and 1 female cadaver bilaterally). The same frequency was observed for the origin from the 1st to 5th costal cartilages (6 of 80 specimens; in 1 male and 1 female cadaver bilaterally, in 1 male and 1 female cadaver unilaterally). In 2.5% of cases (bilaterally in 1 male cadaver), the PM originated from the 1st to 7th costal cartilages.

**Figure 3:**
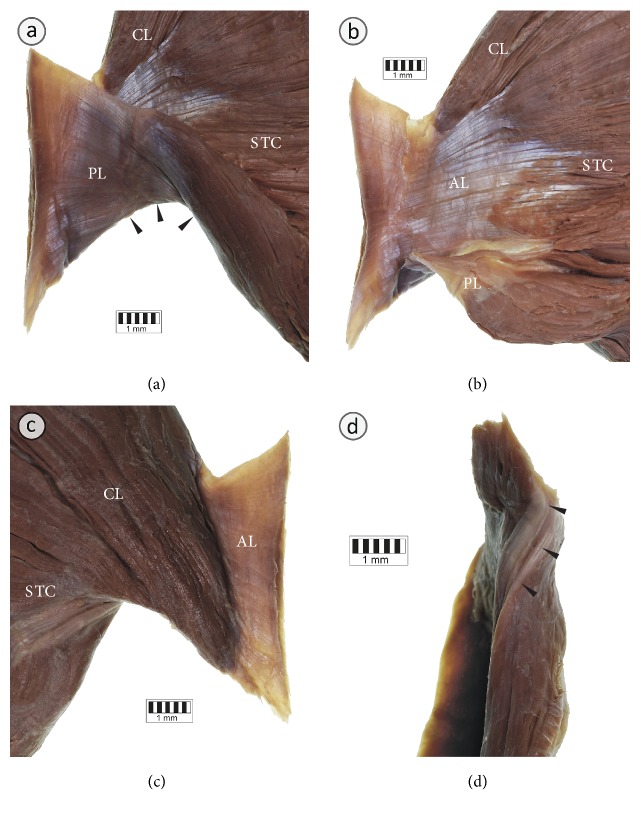
Anatomy of the tendon of the typical pectoralis major muscle. (a) The tendon of the left pectoralis major muscle seen from behind. Posterior lamina (PL) of the pectoralis major muscle tendon constitutes place of attachment for the lower fibers of the sternocostal (STC) part of the muscle. (b) The tendon of the left pectoralis major muscle seen from behind. Posterior lamina (PL) of the pectoralis major muscle has been separated and reflected to expose the posterior view to the anterior lamina (AL) of the tendon. The AL is a place of attachment for the clavicular part (CL), as well as for the upper and middle fibers of the sternocostal (STC) part of the pectoralis major muscle. (c) Anterior view to the tendon of the left pectoralis major muscle. (d) Inferior view to the tendon of the left pectoralis major muscle. Black arrowheads show twisting of the lower fibers of the sternocostal part of the pectoralis major muscle.

**Figure 4:**
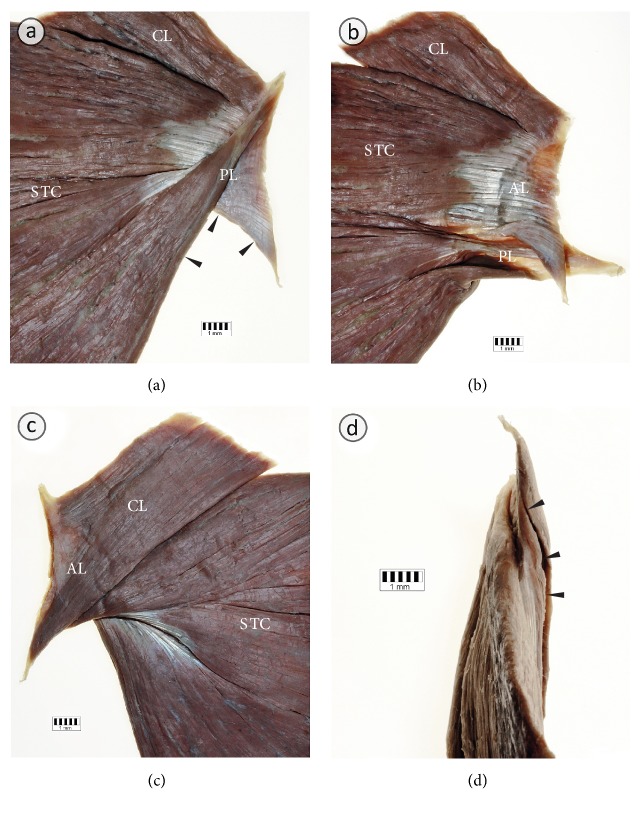
Anatomy of the tendon of the pectoralis major muscle with a separate clavicular part. (a) The tendon of the right pectoralis major muscle seen from behind. Posterior lamina (PL) of the pectoralis major muscle tendon constitutes place of attachment for the lower fibers of the sternocostal (STC) part of the muscle. (b) The tendon of the pectoralis major muscle seen from behind. Posterior lamina (PL) of the pectoralis major muscle has been separated and reflected to expose the posterior view to the anterior lamina (AL) of the tendon. The AL is a place of attachment for the clavicular part (CL), as well as for the upper and middle fibers of the sternocostal (STC) part of the pectoralis major muscle. (c) Anterior view to the tendon of the PM. (d) Inferior view to the tendon of the pectoralis major muscle. Black arrowheads show twisting of the lower fibers of the sternocostal part of the pectoralis major muscle.

**Figure 5:**
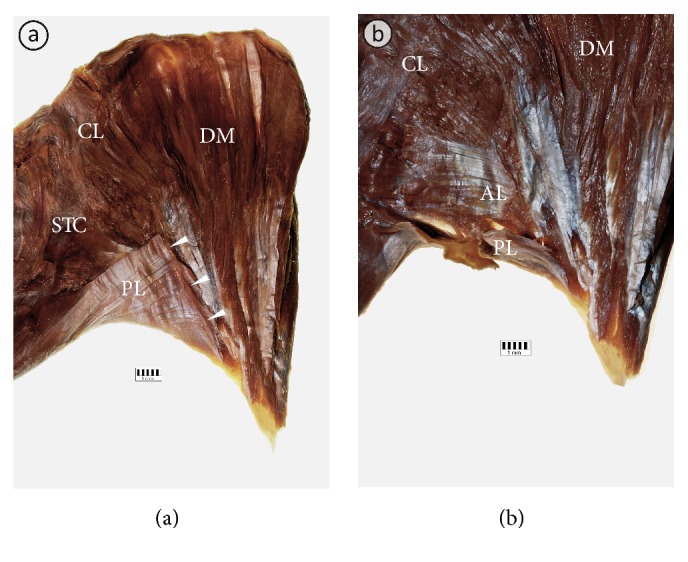
Anatomy of the tendon of the pectoralis major muscle fused with the deltoid muscle. (a) The right pectoralis major muscle and the deltoid muscle seen from behind. Posterior lamina (PL) of the pectoralis major muscle tendon constitutes place of attachment for the lower fibers of the sternocostal (STC) part of the muscle. (b) The tendon of the pectoralis major muscle seen from behind. Posterior lamina (PL) of the pectoralis major muscle has been separated and reflected to expose the posterior view to the anterior lamina (AL) of the tendon. White arrowheads show the insertion of the pectoralis major muscle. CL: clavicular part of the pectoralis major muscle.

**Figure 6:**
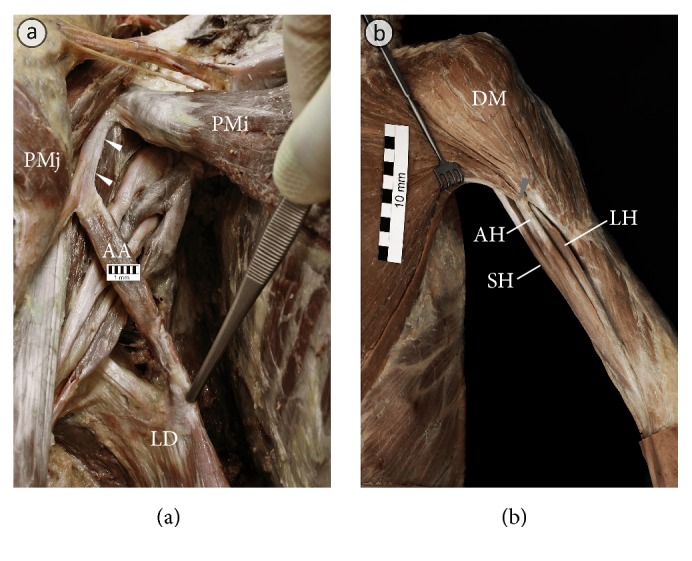
Unusual variations of the tendon of the pectoralis major muscle. (a) An atypical tendinous band (marked by white arrowheads) connecting tendon of the pectoralis major muscle (PMj) with the coracoid process of the scapula. The axillary arch (AA) is stretched between this band and the latissimus dorsi muscle (LD). (b) An accessory head (AH) of the biceps brachii muscle inserted to the tendon of the pectoralis major muscle (place of this insertion is marked by grey arrowhead). DM: deltoid muscle; LH: long head of the biceps brachii muscle; PMi: pectoralis minor muscle; SH: short head of the biceps brachii muscle.

The width of the PM insertion ranged from 43.9 to 83.2 mm (mean = 65.3 mm ± 9.8 mm; Table 3). The distance between the top of the greater tubercle of the humerus and the PM insertion ranged from 38.3 to 65.2 mm (mean = 52.1 mm ± 7.9 mm; Table 3). Anatomy of the insertion of the PM for each anatomical variation is shown in Figures 3–5. The clavicular portion of PM was attached to the anterior lamina of PM insertion in all cases. The posterior lamina of PM insertion was the place of attachment of the sternocostal and abdominal portions. In the case of the fusion between clavicular portions of pectoralis major and deltoid muscles, a close relationship was observed between the posterior lamina of PM insertion and insertion of the deltoid muscle (Figure 5). In 1.25% of specimens (1 of 80 specimens; one male cadaver on the left side) an atypical tendinous band connecting PM insertion with the coracoid process of the scapula was observed (Figure 6(a)). The axillary arch took origin from this band (Figure 6(a)). In another case (1 of 80 specimens; one male cadaver on the left side), the accessory head of the biceps muscle was inserted to the PM tendon (Figure 6(b)).

### 3.3. Observations on the Innervation of PM

**Figure 7:**
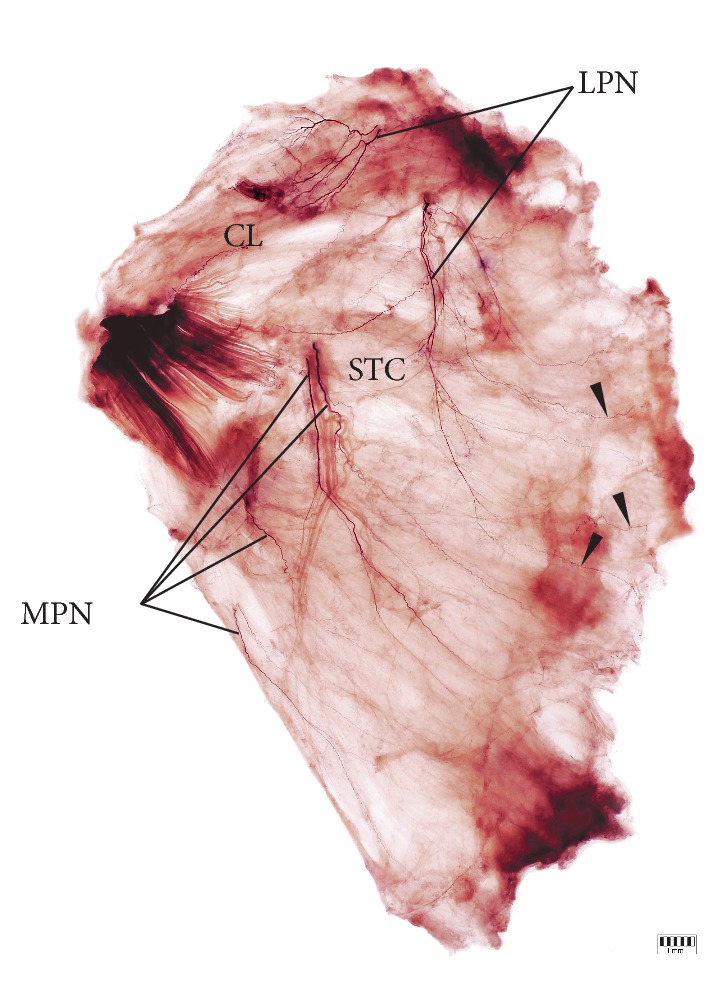
The general pattern of innervation of the lateral and medial pectoral nerves observed on specimen stained by using Sihler's method. The detailed intramuscular distribution of certain nerve sub-branches was exposed. Branches of the intercostal nerves distributed within pectoralis major muscle were marked by black arrowheads. CL: clavicular part of the pectoralis major muscle; LPN: branches of the lateral pectoral nerve; MPN: branches of the medial pectoral nerve; STC: sternocostal part of the pectoralis major muscle.

The detailed intramuscular distribution of certain nerve sub-branches was exposed by Sihler's stain (Figure 7). The general pattern of innervation of the lateral and medial pectoral nerves was observed to be constant. In all specimens of the PM examined in our study the clavicular part of PM was innervated solely by branches of the lateral pectoral nerve (Figure 7). The sub-branches of the lateral pectoral nerve were also distributed within the upper portion of the sternocostal part and they reached the height of approximately the upper half of the muscle (Figure 7). The lower half of PM and the abdominal portion, when present, were innervated by the branches of the medial pectoral nerve. In all specimens stained by Sihler's technique, the contribution of the intercostal nerves in PM innervation was confirmed (Figure 7).

**Figure 8:**
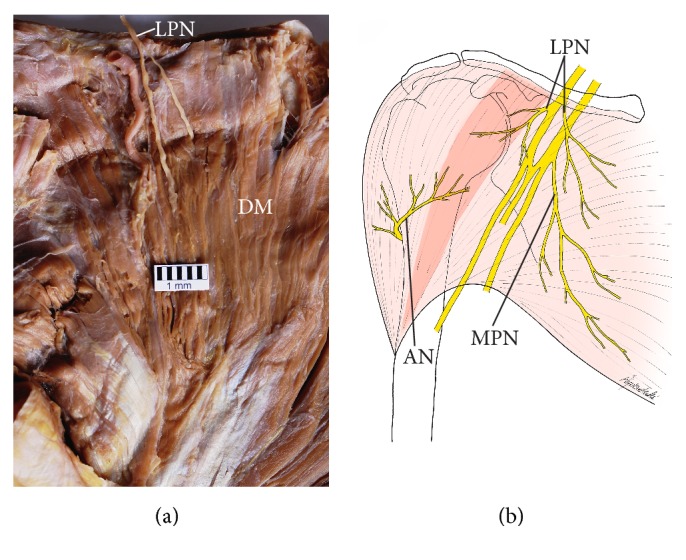
Deviation from the typical distribution of branches of the lateral pectoral nerve observed on the specimen with a complete fusion between the pectoralis major and deltoid muscles. (a) Small sub-branches of the lateral pectoral nerve (LPN) joined the clavicular portion of the deltoid muscle (DM). (b) Schematic representation of the extended territory of the lateral pectoral nerve. AN: axillary nerve; MPN: medial pectoral nerve.

The described pattern was similar in typical specimens and in specimens with a separated clavicular head of PM. In the case of hypotrophy of the clavicular portion of the PM, normal and well-developed branches of the lateral pectoral nerve were observed. Also, as observed by us, a single case of an atypical division of PM into two heads, the upper head (composed of clavicular portion and upper fibers of sternocostal portion of PM) was innervated only by the lateral pectoral nerve, whereas branches of both medial and lateral pectoral nerves innervated the lower head. However, a deviation from the described distribution of branches of the lateral pectoral nerve was observed in one male cadaver with a complete fusion between PM and the deltoid muscle. In this case small sub-branches of the lateral pectoral nerve joined the clavicular portion of the deltoid muscle (Figure 8). Thus, the territory of innervation of the lateral pectoral nerve was extended in this case.

Morphometric characteristics of entry points of the neurovascular pedicles within the PM regarding, respectively, the parasternal line and the inferior border of the clavicle are presented in Tables 4 and 5. The mean distance between the entry points of the medial and lateral pectoral nerves into the PM varied from 38.6 mm to 61.8 mm in male cadavers (mean = 49.7 mm; SD = 11.2 mm). In female cadavers the mean distance between the entry points of the medial and lateral pectoral nerves into the PM varied from 31.2 mm to 60.1 mm (mean = 47.2 mm; SD = 10.6 mm).

## 4. Discussion

### 4.1. Classification of Deviations from the Average Arrangement of the PM and Its Tendon

Perrin [32] already in 1871 suggested a useful and still valid classification of deviations from the average arrangement of the muscles, including, respectively, the presence of not typical muscles; duplication of muscles in whole or in part; fusion of muscles that are typically separate; presence of additional origins, supernumerary tendons or unusual insertions; segmentation (fission) of the muscle; and suppression (partial or complete). Different variations of the PM represent all six classes of anatomical variability of muscles described by Perrin [32].

The example of accessory pectoral muscles which are occasionally present may be pectoralis quartus, pectoralis intermedius, pectoralis minimus, or chondroepitrochlearis [2, 18, 20, 30]. Coexistence of a pectoralis quartus muscle, a supernumerary head of biceps brachii muscle, and an accessory head of flexor digitorum profundus muscle was reported by Song et al. [38]. The presence of supernumerary muscles may potentially affect the surgical procedures. For instance, complicated axillary lymphadenectomy due to a pectoralis quartus muscle was described by Totlis et al. [39]. The presence of the sternalis muscle is observed in 3% to 5% of individuals according to Bergman et al. [2] and in 8% of the population according to Snosek et al. [40]. The sternalis muscle occupies position between the superficial fascia and the pectoral fascia [40]. Different variants of the sternalis muscle were described in medical literature [2, 32, 40, 41]. In our study we found one case of unilateral presence of the sternalis muscle. Our case may be classified as a “simple type” according to Snosek et al. [40] classification system. Davimes et al. [41] suggest that the sternalis muscle may be misinterpreted as a “pathological mass or lesion”; thus clinicians should be aware of this variation during diagnostic procedures. There were also described cases of “duplicity” of the PM. Partial duplicity of the PM was reported by Loukas et al. [23], who observed additional head of PM which fused with the fibers of the serratus anterior muscle. Redler et al. [27] demonstrated a case of anomalous accessory muscle confluent with the normal sternal head of PM. Cases are known of PM fusion with the deltoid muscle [1, 2, 28].

Quinlan et al. [42] pay attention to an unusual humeral insertion of the PM in that the clavicular and upper sternal fibers attach distally on the humerus, while the lower sternal and abdominal fibers cross above the former and insert uppermost on the shaft of the humerus. According to Figueiredo et al. [43] the tendon of the PM presents a single laminar insertion in the humerus. Carey and Owens [44] also proved that it was not possible to differentiate between the two layers of the PM tendon in the region of the insertion in the humerus. These authors found that the mean length of the PM insertion was 72 mm. They also found that the mean distance from the apex of the upper edge of the PM tendon to the superomedial edge of the greater tubercle of the humerus was 42 mm. Figueiredo et al. [43] estimated that the mean proximal to distal border length was 80.8 mm (range: 70–90) and the mean distance from the upper border of the pectoralis major tendon to the apex of the humeral head was 59.3 mm (range: 55–64). The results of our measurements of the PM insertion are similar to the results cited above. In our study, the mean width of the insertion of PM was 65.3 mm (range: 43.9-83.2), while the mean distance between the top of the greater tubercle and upper border of PM tendon was 52.1 mm (range: 38.3-65.2). Fung et al. [45] stated that there is little consensus regarding the complex musculotendinous architecture of the PM. The study of Fung et al. [45] suggested that the muscle belly of the PM consisted of an architecturally uniform clavicular head and a segmented sternal head, while the PM tendon consisted of longer anterior and shorter posterior layers that were continuous inferiorly. Laminar structure of PM tendon observed in our study was constant for all major anatomical variations of the PM, except two unusual cases of an unusual insertion of the PM. In the first case, the insertion of the PM was associated with the presence of the axillary arch and tendinous band attached to the coracoid process of the scapula. In the second, the accessory head of the biceps brachii was attached to the anterior lamina of the PM insertion. The cases of additional origins, supernumerary tendons, or unusual insertions of PM were reported in anatomical literature. Bergman et al. [2] describe occasional occurrence of “additional slip” stretched between PM which extends to biceps, pectoralis minor, the coracoid process, capsule of the shoulder joint, or the brachial fascia. Coexistence of a pectoralis quartus muscle and an unusual axillary arch was described by Bonastre et al. [20].

Bilateral asymmetric deficiency of the pectoralis major muscle was described by Mosconi and Kamath [24]. On the left side, the sternal portion of the sternocostal head of the pectoralis major muscle was absent [24]. On the right side, the entire pectoralis major muscle was absent and the pectoralis minor, deltoid, and coracobrachialis muscles were infiltrated with connective tissue and fat [24]. Cases of congenital absence of PM were also reported on living subjects by Lee and Chun [22], as well as by Mysnyk and Johnson [25]. As Bergman et al. [2] stated, in rare instances the whole PM may be absent; according to estimation of those authors, the muscle was absent in about 0.01% or one in 10,000 individuals. Although the clavicular head is least likely to be absent, in our study the case of hypotrophy of the clavicular part of the PM was observed in one female cadaver.

### 4.2. Clavicular Part of PM and Its Relation to the Deltopectoral Triangle

Typically, the PM is divided into clavicular, manubrial, sternal, and abdominal portions. All these parts may be more or less separable [2]. This especially applies to the clavicular part. The case of clavicular part of the PM separated from the sternocostal part was described by Barberini [19]. Variations of the clavicular part of the PM may be explained and understood based on embryology and phylogeny. As Barberini [19] states, the clavicular part is a new acquisition in Anthropoids. It provides additional stabilization of the upper limb to the thorax thus permitting increased limb mobility [19]. It is also synergetic with the clavicular part of the deltoid muscle.

Because the clavicular part of PM develops from the same origin as deltoid muscle, it remains in close relation to the clavicular part of the deltoid; both parts are connected through fascial structures (deltopectoral fascia), especially in their distal part [46]. The clavicular part of the PM may also extend laterally on the clavicle as far as the deltoid muscle. In those cases, the PM and the deltoid muscle may be fused to varying degrees [2]. The borderline between the two muscles, referred to in clinical jargon as the “deltopectoral interval,” remains an important topographical landmark during various medical procedures such as cephalic vein catheterization or deltopectoral approach for fractures or arthroplasty [9–12]. According to the anatomical nomenclature, the indentation in the muscular structure between the deltoid muscle and PM forms the deltopectoral triangle. Loukas et al. [47] drew attention to anatomical relationships within the deltopectoral triangle. In their study, attention was paid to the fact that deltopectoral triangle may exhibit high variability. In extreme cases, the deltopectoral triangle may not exist as in the case of PM fusion with deltoid muscle, which can potentially cause issues during surgery. A complete fusion between the left pectoralis major and the deltoid muscles, with absence of deltopectoral groove and the infraclavicular fossa, was described by Natsis et al. [26]. The cited authors [26] classified this fusion as “complete fusion into a deltopectoral complex muscle.”

Anatomical variations of the PM may coexist with variations in the course of the cephalic vein [48]. In the presented study in both cases of the total fusion of PM with the deltoid muscle, the anatomical relations between PM and the cephalic vein were altered. In the first cadaver the brachial segment of the cephalic vein was absent; in the second case the cephalic vein pierced the clavicular part of PM to drain into axillary vein. Variable anatomical relations between PM and cephalic vein may be important during surgical procedures. Hong et al. [21] described a case of a cephalic vein which perforated the pectoralis major muscle between the clavicular and sternal heads and then drained into one of the double axillary veins. In the case reported by Hong et al. [21], the cephalic vein was very thin at the lateral arm. The perforating point of the cephalic vein was in those cases located between the clavicular and sternal heads of the pectoralis major muscle. In turn, in the case of fusion between the pectoralis major and the deltoid muscles described by Natsis et al. [26] the cephalic vein and the deltoid branch of the thoracoacromial artery were lying under the fused muscles but had a typical drainage and distribution. Loukas et al. [47] reported the absence of the cephalic vein in 5% of examined specimens. The altered relations between the PM and the cephalic vein may alter medical procedures such as cardiac catheterization, emergency catheterization procedures, or combined use of the cephalic vein and the skin graft-covered pectoralis major muscle flap [21, 47, 49].

### 4.3. General Innervation Pattern of the PM

Wickham et al. [50] described anatomical and functional segmentation of selected shoulder joint musculature. Moreover, research on the innervation of selected muscles suggests that axons migrating during development into effector organs show a well-defined specificity [37]. Such specificity may also occur in relation to the thoracic nerves and segments of PM innervated by them. Our study, supplemented by using Sihler's stain technique, is in accordance with previous reports on the segmented innervation pattern of the pectoral nerves [19]. Both in our study and in cases described in the literature, the medial pectoral nerve innervates solely the lower PM segments independently of the anatomical variation. In contrast, the lateral pectoral nerve is involved in the innervation of the clavicular portion and the upper segments of the sternocostal portion. A relatively morpho-functional independence of the clavicular part of the rest of the PM was discussed by Barberini [19], who suggested that the width of the lateral pectoral nerve, which supplies the clavicular part of the muscle, may be related to a greater functional ability. In our study, well-developed lateral pectoral nerves were observed independently of the PM variation, even in the case of a significantly reduced clavicular portion. Thus, although the pectoral nerves are characterized by high anatomical variability according to their origin and course [51, 52], their territory seems to be constant. Such observations coincide with information provided by Bergman et al. [2] who noted the occurrence of the lateral pectoral nerve even with congenital PM deficiency. However, some authors reported lack of selected pectoral nerves related to defects of the PM. For instance, Yamasaki [31] reported two cases of the congenital partial defect of pectoralis major and minor muscles. In both cases described by Yamasaki [31], the PM was defected, with the clavicular portion and a small part of sternocostal portion only persisting. Both the lateral and medial pectoral nerves supplied the pectoral muscles in the first case and medial pectoral nerve was distributed unusually to the most lateral part of the persisted sternocostal portion [31]. Only the lateral pectoral nerve existed in the second case [31]. In turn, in the case of asymmetric deficiency of the pectoralis major muscle described by Mosconi and Kamath [24], on both sides, the lateral pectoral nerves were absent and the medial pectoral nerves were present.

However, small deviations of pectoral nerves in the territory of the deltoid muscle were reported. Anatomical variant of the lateral pectoral nerve innervating the anterior portion of the deltoid muscle was documented by Solomon et al. [28]. Similar case was found in our study and was associated with the fusion between the clavicular part of the PM and the deltoid muscle. Also intercostal nerves may participate in PM innervation. In Beheiry's [53] study, the fourth intercostal nerve participated in supply of the inferolateral part of the PM in 4 out of 30 cases. In our study all specimens stained by Sihler's technique showed the contribution of the intercostal nerves in PM innervation. However, the character of fibers provided to PM by intercostal nerves (motor or/and sensory) could not be determined basing on macroscopic methods. Due to the fact that numerous procedures for plastic and reconstructive surgery are performed by isolating the clavicular part of the PM [6–8, 19, 53–55], knowledge of both innervation pattern of the PM and its anatomical variations may be important from the clinical point of view. 

Also the knowledge of the entry points and course of the neurovascular pedicles may be crucial for the surgery of the PM. The clavicular head of the PM and the superior part of the sternal head of the muscle are innervated by the lateral pectoral nerve. Wei and Chan [56] state that this nerve is characterized by a constant course. According to those authors, the nerve courses on the deep surface of the PM for a mean of 55 ± 7 mm and is visible under the muscle [56]. The medial pectoral nerve, in turn, supplies the posterolateral parts of the sternal head of the PM. This nerve has a more variable course, piercing and supplying the pectoralis minor at the level of the third intercostal space, at a mean of 10.3 cm from the margin of the sternum [56]. According to Wei and Chan [56], the mean distance between the entry of the medial and lateral pectoral nerves into the PM is 30.7 ± 10 mm. However, in our study the mean distance between the entry of the medial and lateral pectoral nerves into the PM was greater and varied between 31.2 mm and 61.8 mm.

## 5. Limitations of the Study

Because the arteries were not injected by the resin, we were not able to trace detailed distribution of arterial branches within the PM. Further studies should be carried out in this regard. When the arteries are injected by the resin, the initial part of Sihler's method (destaining) may be used to trace detailed intramuscular arterial pattern. However, to avoid altering of nerves staining and visualization, the study on distribution of arteries should be performed separately.

## 6. Conclusions

The general pattern of innervation of the lateral and medial pectoral nerves was observed to be constant. PM is mainly innervated by the lateral pectoral nerve. Surgeons should be aware of anatomic variations of the PM both in planning and in conducting surgeries of the pectoral region.

## Figures and Tables

**Table 1:** The incidence of different types of anatomical variations of the pectoralis major muscle (PM).

Type of anatomical variation	Incidence [%] in male cadavers (n = 22)	Incidence [%] in female cadavers (n = 18)	Total incidence [%] in examined cadavers (n = 40)	Total incidence [%] for all examined specimens (n = 80)
Typical morphology of PM	Bilaterally: 13 cadavers (13/22 = 59.1%); Unilaterally: 1 cadaver (1/22 = 4.5%)	Bilaterally: 12 cadavers (12/18 = 66.7%)	Bilaterally: 25 cadavers (25/40 = 62.5%); Unilaterally; 1 (1/40 = 2.5%)	51 specimens (51/80 = 63.75%)

Separation of clavicular portion of PM	Bilaterally: 6 cadavers (6/22 = 27.3%)	Bilaterally: 5 cadavers (5/18 = 27.7%)	Bilaterally: 11 cadavers (11/40 = 27.5%)	22 specimens (22/80 = 27.5%)

Atypical division of PM (clavicular portion fused with upper fibers of sternocostal portion)	Unilaterally: 1 cadaver (1/22 = 4.5%)	-	Unilaterally: 1 cadaver (1/40 = 2.5%)	1 specimen (1/80 = 1.25%)

Hypotrophy of clavicular portion of PM	-	Bilaterally: 1 cadaver (1/18 = 5.6%)	Bilaterally: 1 cadaver (1/40 = 2.5%)	2 specimens (2/80 = 2.5%)

Fusion between the clavicular portion of PM and the deltoid muscle	Complete (absence of deltopectoral groove): 1 cadaver (1/22 = 4.5%); Partial (deep fibers fused): 1 cadaver (1/22 = 4.5%) Total: 2 cadavers (2/22 = 9.1%)	-	Complete (absence of deltopectoral groove): 1 cadaver (1/40 = 2.5%); Partial (deep fibers fused): 1 cadaver (1/40 = 2.5%) Total: 2 cadavers (2/40 = 5%)	Complete (absence of deltopectoral groove): 2 specimens (2/80 = 2.5%); Partial (deep fibers fused): 2 specimens (2/80 = 2.5%); Total incidence of fusion: 4 specimens (4/80 = 5%)

Presence of the sternalis muscle	-	Unilaterally: 1 cadaver (1/18 = 5.6%)	Unilaterally: 1 cadaver (1/40 = 2.5%)	1 specimen (1/80 = 1.25%)

**Table 2:** Morphometric characteristics of the anatomical variations of the clavicular part of the pectoralis major muscle (PM).

*The width of the origin of the clavicular part of PM [mm]*						
		Minimal value	Maximal value	Mean	Median	Standard deviation

Typical variants of PM	Male cadavers	70	105	87	83	13
	Female cadavers	63	88	76	77	9
	*Total*	*63*	*105*	*82*	*81*	*12*

Variants with separated clavicular part	Male cadavers	47	92	69	70	18
	Female cadavers	59	84	72	73	10
	*Total*	*47*	*92*	*66*	*68*	*17*

Variants with fusion between PM and deltoid muscle	Observed only in two male cadavers	99	115	106	105	7

Atrophy of the clavicular part of PM	Observed only in one female cadaver	32	38	35	35	4

*Percentage of total length of the clavicle covered by the origin of the clavicular part of PM [%]*						

		Minimal value	Maximal value	Mean	Median	Standard deviation

Typical variants of PM	Male cadavers	47.2	68.7	56.9	52.6	9.4
	Female cadavers	42.5	79.2	57.1	50.9	13.1
	*Total*	*42.5*	*79.2*	*57*	*51*	*11*

Variants with separated clavicular part	Male cadavers	31.9	56.4	40.4	42.5	9.9
	Female cadavers	40.1	50.9	46.2	47,2	3.8
	*Total*	*31.9*	*56.4*	*43.5*	*43.6*	*7.6*

Variants with fusion between PM and deltoid muscle	Observed only in two male cadavers	60.1	83.3	71.5	70.4	12.6

Atrophy of the clavicular part of PM	Observed only in one female cadaver	22.5	26	24.2	24.2	2.5

*Asymmetry between right and left side in percentage of total length of the clavicle covered by the origin of the clavicular part of PM [%]*						

		Minimal value	Maximal value	Mean	Median	Standard deviation

Typical variants of PM	Male cadavers	0.9	23.2	8.4	6.7	7.8
	Female cadavers	4.3	11.2	7.4	7.2	2.6
	*Total*	*0.9*	*23.2*	*7.9*	*7.2*	*5.8*

Variants with separated clavicular part	Male cadavers	1.1	69.9	28.2	23	26.9
	Female cadavers	6.1	10.8	7.8	7.2	2.3
	*Total*	*1.1*	*69.9*	*19.1*	*10.2*	*21.9*

Variants with fusion between PM and deltoid muscle	Observed only in two male cadavers	0.3	3.5	1.9	1.9	2.3

Atrophy of the clavicular part of PM	Observed only in one female cadaver	Asymmetry = 15.6%				

*The width of the base of the deltopectoral triangle (i.e. the distance between origins of the clavicular parts of PM and the deltoid muscle) [mm]*						

		Minimal value	Maximal value	Mean	Median	Standard deviation

Typical variants of PM	Male cadavers	10.3	20.9	16.2	17.3	3.8
	Female cadavers	15.3	30.8	23	22.9	6.6
	*Total*	*10.3*	*30.8*	*20.2*	*18.5*	*6.4*

Variants with separated clavicular part	Male cadavers	13.4	38.6	20.5	19.3	7.4
	Female cadavers	11.5	35.6	21.9	18.5	8.1
	*Total*	*11.5*	*38.6*	*21.3*	*18.9*	*7.6*

Variants with fusion between PM and deltoid muscle	Observed only in two male cadavers	0	0	0	0	0

Atrophy of the clavicular part of PM	Observed only in one female cadaver	49.8	52.9	51.4	51.4	2.2

**Table 3:** Morphometric characteristics of the pectoralis major muscle (PM).

*The width of the origin of the sternocostal part of PM [mm]*					
	Minimal value	Maximal value	Mean	Median	Standard deviation

Male cadavers	160	215	182	185	17

Female cadavers	133	181	156	154	19

*Total*	*133*	*215*	*172*	*177*	*22*

*The width of the PM in the midclavicular line (measured to the level of inferior border of the muscle) [mm]*					

	Minimal value	Maximal value	Mean	Median	Standard deviation

Male cadavers	172	224	196	194	16

Female cadavers	173	223	194	191	16

*Total*	*172*	*224*	*196*	*194*	*15*

*Asymmetry of the width of the PM in the midclavicular line [%]*					

	Minimal value	Maximal value	Mean	Median	Standard deviation

Male cadavers	1.6	17.9	7.3	3.4	6.2

Female cadavers	1.4	12.1	6.4	6.3	4

*Total*	*1.4*	*17.9*	*6.9*	*5.2*	*5.2*

*The width of the insertion of PM [mm]*					

	Minimal value	Maximal value	Mean	Median	Standard deviation

Male cadavers	53.8	83.2	66.7	66	9.7

Female cadavers	43.9	78.3	62.9	60.3	10.2

*Total*	*43.9*	*83.2*	*65.3*	*63.6*	*9.8*

*The distance between the top of the greater tubercle and upper border of PM tendon [mm]*					

	Minimal value	Maximal value	Mean	Median	Standard deviation

Male cadavers	38.3	65.2	53.9	54.2	8.2

Female cadavers	41.5	58.5	48.1	47.9	5.8

*Total*	*38.3*	*65.2*	*52.1*	*51.8*	*7.9*

**Table 4:** Morphometric characteristics of entry points of the neurovascular pedicles within the pectoralis major muscle (PM) regarding the parasternal line.

Distance from the margin of the sternum (parasternal line) to the:	Min [mm]	Max [mm]	Mean [mm]	Median [mm]	SD [mm]
*Male cadavers (n = 22)*					

entry points of the LPN to the clavicular part of PM	80.1	113.9	95.4	94.4	12.8

entry points of the LPN to the sternocostal part of PM	104.2	121.8	113.5	111.7	6.5

entry points of the MPN to the sternocostal part of PM	97.2	121.5	112.7	115.8	9.1

entry points of the MPN to the abdominal part of PM	110.1	134.8	120.7	119.4	8

*Female cadavers (n = 18)*					

entry points of the LPN to the clavicular part of PM	48.9	79.1	67.1	67	10.6

entry points of the LPN to the sternocostal part of PM	84.5	101.7	91.2	89.2	6.5

entry points of the MPN to the sternocostal part of PM	85.5	107.8	100.1	102.2	7.4

entry points of the MPN to the abdominal part of PM	86.9	122.3	108.5	105.7	10.9

*Total (n = 40)*					

entry points of the LPN to the clavicular part of PM	48.9	113.9	83.2	80.2	18.4

entry points of the LPN to the sternocostal part of PM	84.5	121.8	103.6	104.2	12.9

entry points of the MPN to the sternocostal part of PM	85.5	121.5	106.4	104.9	10.4

entry points of the MPN to the abdominal part of PM	86.9	134.8	112.9	114	11.6

LPN: lateral pectoral nerve; MPN: medial pectoral nerve.

**Table 5:** Morphometric characteristics of entry points of the neurovascular pedicles within the pectoralis major muscle (PM) regarding the inferior border of the clavicle.

Distance from the inferior border of the clavicle to the:	Min [mm]	Max [mm]	Mean [mm]	Median [mm]	SD [mm]
*Male cadavers*					

entry points of the LPN to the clavicular part of PM	18.8	40.1	27.7	24.6	7.4

entry points of the LPN to the sternocostal part of PM	62.8	91.6	71.4	75.8	15.3

entry points of the MPN to the sternocostal part of PM	104.2	123.6	114.7	115.3	6.4

entry points of the MPN to the abdominal part of PM	110,1	140,6	121.8	120.7	12.8

*Female cadavers*					

entry points of the LPN to the clavicular part of PM	17.1	39.6	27.6	30.2	8.6

entry points of the LPN to the sternocostal part of PM	61.5	81.4	70.3	70.5	5.6

entry points of the MPN to the sternocostal part of PM	79.8	119.4	102.7	104.3	13.4

entry points of the MPN to the abdominal part of PM	102.3	129.8	112.7	110	10.1

*Total*					

entry points of the LPN to the clavicular part of PM	17.1	40.1	27.7	27.4	8.2

entry points of the LPN to the sternocostal part of PM	61.5	91.6	70.9	71.2	11.8

entry points of the MPN to the sternocostal part of PM	79.8	123.6	108.3	110.1	12.3

entry points of the MPN to the abdominal part of PM	102.3	140.6	117.7	117.6	12.5

LPN: lateral pectoral nerve; MPN: medial pectoral nerve.

## Data Availability

The data used to support the findings of this study are available from the corresponding author upon request.
